# Induction chemotherapy followed by neoadjuvant chemoradiotherapy and surgery in locally advanced rectal cancer: preliminary results of a phase II study

**DOI:** 10.18632/oncotarget.26101

**Published:** 2018-09-14

**Authors:** Francesca De Felice, Giancarlo D’Ambrosio, Daniela Musio, Franco Iafrate, Ilaria Benevento, Marco Marzo, Marialaura Mancini, Federica Urbano, Marcella Iannitti, Francesco Marampon, Nadia Bulzonetti, Enrico Cortesi, Vincenzo Tombolini

**Affiliations:** ^1^ Department of Radiological Sciences, Oncology and Pathology, Policlinico Umberto I, “Sapienza” University of Rome, Rome, Italy; ^2^ Department of General Surgery, Policlinico Umberto I, “Sapienza” University of Rome, Rome, Italy

**Keywords:** rectal cancer, induction chemotherapy, mutation, chemoradiotherapy, complete response

## Abstract

**Background and purpose:**

To report preliminary results of induction chemotherapy (IC) followed by neoadjuvant chemoradiotherapy (CRT) and surgery in locally advanced rectal cancer (LARC) patients.

**Materials and methods:**

This is the preliminary evaluation of a phase II study. Patients with histologically proven rectal adenocarcinoma, stage II-III disease, who met the inclusion criteria, received induction FOLFOXIRI (5-FU, leucovorin, oxaliplatin and irinotecan) regimen in combination with targeted agents followed by CRT and surgery. Analysis of the first 8 patients was required to confirm the treatment feasibility before the accrual of 20 additional patients.

**Results:**

The first 8 patients were evaluated. The median follow-up time was 23 months. There were no treatment-related deaths. Trimodality strategy was well tolerated with high compliance and a good level of toxicity. There were no evidence of febrile neutropenia and any grade 4 adverse events were recorded. Three patients had pathologic complete response (pCR) and 1 patient had a nearly pCR (ypT1 ypN0).

**Conclusion:**

Preliminary results are encouraging. FOLFOXIRI regimen plus targeted agents followed by CRT and surgery seems a safe approach. Longer follow-up and higher number of patients are mandatory to confirm such findings.

## INTRODUCTION

Despite validation of the trimodality treatment approach, including neoadjuvant 5-fluorouracil (5-FU)-based chemoradiotherapy (CRT), surgery and adjuvant chemotherapy, distant metastasis remains a devastating issue of failure in locally advanced rectal cancer (LARC) management [1].

A recent meta-analysis concerning 3310 patients involved in four randomized trials showed a modest distant failure benefit (OR = 0.76; 95% CI, 0.60 to 0.97; p = 0.03) in adding oxaliplatin compared to standard 5-FU-based CRT [2]. But, definitive conclusions are still pending and the frequent development of distant metastasis in LARC continues to stimulate research community attention. The main consideration is the time window of approximately 4 months from neoadjuvant CRT to systemic chemotherapy which may potentially facilitate distant micrometastasis growth. Shifting systemic chemotherapy as initial approach into the trimodality treatment paradigm could represent a tangible option to target micrometastasis. Compared with postoperative CAPOX (capecitabine and oxaliplatin) regimen, induction CAPOX before neoadjuvant CRT achieved a more favorable compliance and toxicity profiles with similar pathologic complete response (pCR) rate, in several retrospective analysis and phase II randomized trials [3–4]. But these studies were not powered to draw any conclusions on survival outcomes. Therefore the risk of systemic spread remains a challenge.

Over the past decade, literature data reflects also a growing interest in defining a more effective systemic cytotoxic therapy. Year by year, Ras (KRas and NRas) and BRAF mutation status detection has become a crucial prognostic and predictive factor in colorectal patients [5–6]. Nowadays, the national comprehensive cancer network (NCCN) recommends tumor tissue genotyped for Ras-BRAF mutations in suspected or metastatic rectal cancers in order to tailor therapy and to confer – if any – benefit to patients [1].

Based on NCCN rectal guidelines and on the efficacy of the FOLFOXIRI (5-FU, leucovorin, oxaliplatin and irinotecan) regimen in combination with targeted agents demonstrated in wild-type KRas colorectal cancer patients [1, 7], we tested the utility of induction chemotherapy (IC) preceding neoadjuvant CRT and surgery resection in LARC. We report the preliminary results of a phase II study in order to provide an early opportunity to evaluate the efficacy of this new treatment sequence.

## RESULTS

### Patient characteristics

The first 8 patients were accrued between October 2015 and September 2016. Table 1 lists the demographics of the study population. In total, 5 patients were classified as cT4 and 4 patients had tumor in the low rectum. There was only 1 patients with wild-type Ras-BRAF tumor and he was treated with FOLFOXIRI regimen combined with panitumumab. The remainder (n = 7) received FOLFOXIRI regimen combined with bevacizumab.

**Table 1:** Patient characteristics

Characteristic	n (%)
Age (years)	
median (range)	66 (44-70)
Gender	
Male	5 (62.5)
Female	3 (37.5)
Smoke	
Yes	6 (75)
No	2 (25)
Comorbidity	
none	5 (62.5)
Arterial hypertension	3 (37.5)
Clinical tumor stage (T)	
T1-2	0
T3	3 (37.5)
T4	5 (62.5)
Clinical nodal stage (N)	
N0	0
N1	2 (25)
N2	6 (75)
Location from anal verge	
< 6 cm	4 (50)
6 - 8 cm	4 (50)
> 8 cm	0

### Induction chemotherapy compliance and toxicity

Globally, 7 patients completed the planned cycles of IC. One patient underwent sudden cardiac death between the first and second cycle. The event was judged not related to study procedure. Patient history revealed arterial hypertension. In total, IC treatment was delayed because of toxic effect in one patient. Overall, grade 2 peripheral neurotoxicity (n = 2) and grade 3 neutropenia (n = 3) were observed. There were no evidence of febrile neutropenia and any grade 4 adverse events were recorded. The only other grade 3 toxic effects observed were oral mucositis (n = 1) and hyperkalemia (n = 1). No patients experienced grade 3 diarrhea.

### Neoadjuvant chemoradiotherapy compliance and toxicity

In total, 7 patients completed the programmed neoadjuvant CRT. As mentioned previously, one patient died during IC because of unrelated treatment cancer conditions. All patients received the RT prescribed total dose and no treatment interruptions were recorded. Due to IC-related toxicity, 4 patients received standard 5-FU-based concomitant chemotherapy. No patients suspended chemotherapy definitely, and thus they received at least 5 cycles of 5-FU with/without oxaliplatin. Acute toxicity incidences during IC and during CRT are shown in Table 2. Interestingly, there were no severe acute complications during neoadjuvant CRT.

**Table 2:** Acute toxicity in preoperative phase

Acute toxicity	IC		Neoadjuvant CRT		Global	
	G1-2 (%)	G3-4 (%)	G1-2 (%)	G3-4 (%)	G1-2 (%)	G3-4 (%)
Allergy immunology						
Allergic reaction hypersensitivity	-	-	-	-	-	-
Constitutional symptoms						
Fatigue	4 (50)	-	3 (37.5)	-	7 (87.5)	-
Fever	1 (12.5)	-	3 (37.5)	-	4 (50)	-
Palpitation	-	-	-	-	-	-
Dermatology skin						
Rash desquamation	2 (25)	-	2 (25)	-	4 (50)	-
Radiation dermatitis	-	-	2 (25)	-	2 (25)	-
Gastrointestinal						
Disgeusia	2 (25)	-	-	-	2 (25)	-
Oral mucositis	-	1 (12.5)	-	-	-	1 (12.5)
Constipation	1 (12.5)	-	2 (25)	-	3 (37.5)	-
Diarrhoea	2 (25)	-	1 (12.5)	-	3 (37.5)	-
Nausea	4 (50)	-	-	-	4 (50)	-
Vomiting	-	-	-	-	-	-
Proctitis	-	-	2 (25)	-	2 (25)	-
Haemorrhage rectum	2 (25)	-	-	-	2 (25)	-
Metabolism disorders						
Hyperkalemia	-	1 (12.5)	-	-	-	1 (12.5)
ALT/AST increased	2 (25)	-	-	-	2 (25)	-
Neurology						
Neuropahy: sensory	4 (50)	-	2 (25)	-	6 (75)	-
Pain						
Abdominal pain or cramping	-	-	-	-	-	
Renal genitourinary						
Dysuria	-	-	1 (12.5)	-	1 (12.5)	-
Urinary frequency	-	-	1 (12.5)	-	1 (12.5)	-
Blood count						
Neutropenia	2 (25)	3 (37.5)	-	-	2 (25)	3 (37.5)
Haemoglobin	-	-	1 (12.5)	-	1 (12.5)	-

Abbreviations: IC: induction chemotherapy; CRT: chemoradiotherapy; G: grade; ALT/AST: alanine transaminase/ aspartate transaminase.

### Trimodality treatment efficacy

**Figure 1:**
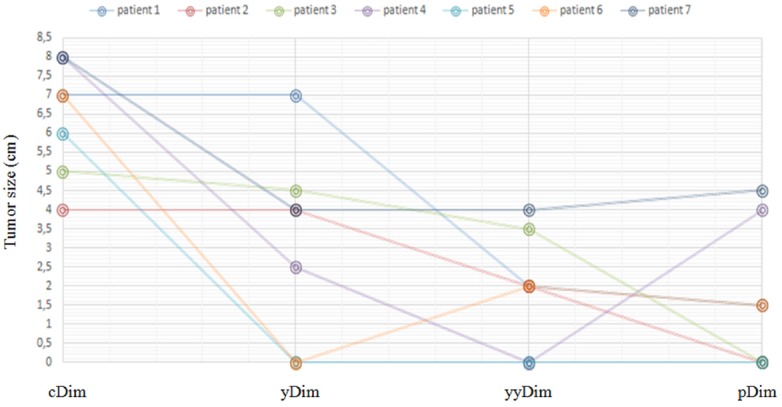
Tumor size evaluation Tumor size at diagnosis (cDim), after induction chemotherapy (yDim), after neoadjuvant chemoradiotherapy (yyDim) and detected in the operative specimen (pDim).

**Figure 2:**
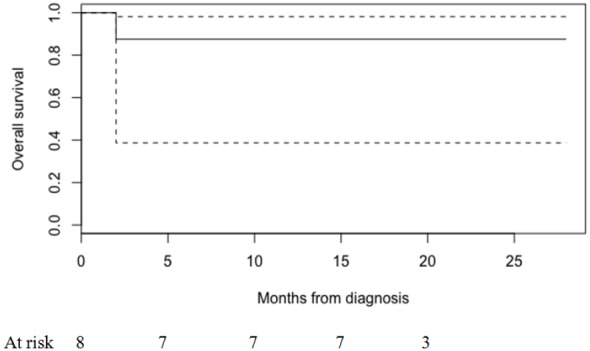
Overall survival

After the end of CRT, four patients had their tumor shrinked more than 50% of the original measurement. Tumor size evaluation at diagnosis (cDim), post-IC (yDim), post CRT (yyDim) and detected in the operative specimen (pDim) is demonstrated in Figure 1. A clinical complete response was noted in one patient, whereas clinical tumor reevaluation was stable in 2 cases. Conservative surgery was performed in five patients and the remainder (n = 2) had a Miles surgery due to closeness to the anal verge. In total, 3 patients had pCR and 1 patient had a nearly pCR (ypT1 ypN0). None had positive radial margins. No peri-operative complications occurred. The median follow-up period was 23 months (range 18-28). At the time of the analysis, there were no treatment-related deaths. The estimated 1-year overall survival was 87.5% (0.387 - 0.981) (Figure 2). Among the 7 patients alive there was no initial evidence of local recurrence disease. One patient developed a distant relapse to the liver six months after surgery.

## DISCUSSION

In the first stage of this two-stage design, 3 of the first 8 patients had pathologic complete response (pCR), which allowed the recruitment of 20 additional patients for the second stage. Globally, IC was well tolerated without negative impact – in term of interruption or major toxicities – on subsequent neoadjuvant CRT and resection. IC was given for up to four two-week cycles. There were no grade 4 toxicities and no disease progression was seen during preoperative treatment phase. R0 resection was achieved in all patients who underwent surgery. To our knowledge, our experience represents the first series testing IC based on FOLFOXIRI regimen combined with targeted agents in LARC.

In the metastatic setting for colorectal cancer, FOLFOXIRI plus panitumumab or bevacizumab has shown promising clinical activity in Ras–BRAF wild-type patients [7, 8]. The combination of triplet chemotherapy and panitumumab or bevacizumab has shown equivalent or superior safety profile, as well as significant improvement in tumor response compared with the available literature data for two cytotoxic drugs (a fluoropyrimidine plus either oxaliplatin or irinotecan) with or without biologics [7]. Therefore, it was rational for us to investigate FOLFOXIRI plus targeted agents as systemic chemotherapy in LARC. Moreover, considering that pCR has been shown to be an efficacious surrogate endpoint for survival outcomes in LARC [9–10], understanding the effect of chemotherapy intensification, using FOLFOXIRI plus targeted agents, in a preoperative setting could be essential to properly improve pCR rate. A phase II clinical trial was designed to provide an early opportunity to evaluate the efficacy of this new strategy treatment. It serves as a platform to highlight opportunities to change LARC approach drug, with the goal to improve survival outcomes. Preliminary data were encouraging, although second stage is mandatory to interpret final results and to appropriately translate them into clinical practice.

There are several considerations that should be made. Firstly, an earlier intervention with systemic treatment could facilitate full dose chemotherapy administration and tumor regression. It has been estimated that in more than 30% of LARC patients adjuvant chemotherapy is omitted or is dose reduced, increasing the risk of systemic failures [10]. Theoretical advantage of IC may allow improvement of oxygenation and higher intramural concentration of cytotoxic drugs to the primary tumor. Currently, Ras-BRAF became a high-priority target also in advanced rectal patients given the prevalence of mutations in rectal adenocarcinoma [11]. In our study, testing Ras-BRAF mutation status was paramount in driving therapeutic decisions. Cetuximab and panitumumab – two anti-epidermal growth factor receptor (EGFR)- targeted antibodies – are effective in wild-type disease [12–13]. Patients with known Ras mutations had virtually no chance of benefit from anti-EGFR agents and thus they were not treated with cetuximab and panitumumab. In fact, exposure to anti-EGFR agents toxicities would not be justified. Bevacizumab, an anti-angiogenic agent, was administred in Ras-BRAF mutated patients, because its efficacy did not depend on the Ras-BRAF mutational status [11].

Secondly, our patient population, including all cases with cT3-4 disease and/or positive nodes but high-lying cT3N0, is representative of a poor prognosis group. At diagnosis, all patients underwent pelvic DW-MRI and definition of poor prognosis disease was based on several parameters, including clinical stage, size, distance from anal merge, sphincter infiltration, potential mesorectal fascia involvement and extra-mural or venous invasion. Each of these parameters allowed an improved prediction of worse outcome, reinforcing the fact that an aggressive systemic strategy could show promising clinical benefits in this setting of patients [14–16]. Our main worry was that patients would be not able to tolerate subsequent CRT. Actually, all patients received neoadjuvant CRT without interruption or dose-limiting toxicity.

Lastly, in LARC, no data are available about the association between IC and survival outcomes after the introduction of targeted agents. FOLFOXIRI plus targeted agents was repeated every 14 days for up to 8 cycles (four two-week cycles). Short induction period (4 months) was selected in order to explore the benefit from intensification of neoadjuvant CRT in the sequential strategy.

At present, we were unable to address LF, DF and median OS due to the short interval follow-up. Moreover, patients cohort was too small to provide firm conclusions. As a results of a favorable pCR profile in the first stage of the two-stage design, the trial would continue to the second stage with the accrual of additional patients.

## MATERIALS AND METHODS

This phase II study is a research project coordinates by Department of Radiological Sciences, Oncology and Pathology, Policlinico Umberto I “Sapienza” University of Rome and it was approved by the” Sapienza” University of Rome (number 88569-140). All patients provided written consent to participate. The preliminary evaluation of the first 8 patients was required to confirm the feasibility of the treatment before completing the enrollment of additional 20 patients.

### Patient selection

Selection criteria included patients with newly diagnosed histologically proven rectal adenocarcinoma within 12 cm from the anal verge, staged T3-4 tumor and/or positive lymph-nodes, without any evidence of distant metastases; age ≥ 18 and ≤ 70 years; performance status ≤ 2; adequate renal, hepatic and bone marrow function. Patients with high-lying cT3N0 disease were not included. Patients were also excluded in case of synchronous tumors, cardiovascular disease, history of neurological or psychiatric disorders, previous pelvis radiotherapy, contraindication to MRI examination.

Clinical examinations, including complete medical history and careful physical examination, as well as digital rectal examination, were combined with radiologic imaging to assess the precise local (T), regional nodal (N), and distant (M) extent of the tumor. Radiologic imaging consisted of trans-rectal ultrasound, total body contrast-enhanced computed tomography (CT) and pelvic diffusion-weight magnetic resonance imaging (DW-MRI). Ras and BRAF mutational analyses was carried out on tumor biopsy.

### Study treatment

All patients were treated with a multimodal treatment approach combining IC, followed by intensified neoadjuvant CRT and surgery.

IC consisted of four two-week cycles of the three-drug regimen of leucovorin-modulated 5-fluorouracil, irinotecan, and oxaliplatin (FOLFOXIRI). Modifications have been made to standard FOLFOX with goals of increasing efficacy. Moreover, based on Ras-BRAF status, biologic agents, including bevacizumab, panitumumab or cetuximab, were added. Only patients with wild-type KRas genes received treatment with cetuximab or panitumumab. Due to potentially severe toxicity, patients aged older than 70 years were excluded from the trial.

Therefore, IC consisted of four two-week cycles of the FOLFOXIRI regimen: irinotecan 165 mg/m^2^ day 1, oxaliplatin 85 mg/m^2^ day 1, leucovorin 200 mg/m^2^ day 1, fluorouracil 2400 mg/m^2^ 48-hour continuous infusion starting on day 1. In addition, cetuximab (400 mg/m^2^ first infusion, 250 mg/m^2^ thereafter) or panitumumab (6 mg/kg) or bevacizumab (5 mg/kg) were administered intravenously based on wild-type (cetuximab/panitumemab) or mutated (bevacizumab) Ras-BRAF status. IC was left to the oncologists’ discretion, because of individual variations in the patient conditions and medical circumstances.

Two weeks from the end of last IC cycle, the assessment of local clinical response was performed by pelvic DW-MRI. Independent of clinical response, long course of intensified CRT was started within four weeks after IC completion. The detailed CRT protocol has been described previously [17–18]. Radiation therapy was delivered with intensity modulate technique at a dose of 45 Gy (1.8 Gy/fr) to the whole pelvis plus 5.4/9 Gy (1.8 Gy/fr) to the tumor volume with 6 to 15 MV energy photons. Concomitant chemotherapy consisted of OXP (50 mg/m^2^ on the first day of each week of RT) and 5-FU (200 mg/m^2^/5 daily continuous infusion).

Five weeks from the end of CRT, the assessment of local clinical response was performed by pelvic DW-MRI. Surgery was scheduled 7 to 9 weeks after the end of CRT treatment.

### Study end point and statistics

Primary end point was the number of patients with pCR. pCR was defined as the absence of any residual tumor cells (ypT0) detected in the operative specimen, including the primary tumor area, the whole mesorectal fat, and the resected lymph nodes (ypN0).

Secondary end-points included toxicity and compliance with the regimen, tumor downstaging, R0 resection rate, surgical complications, sphincter preservation rate, local and distant failure rates, overall survival (OS). The assessment of local clinic response was performed by pelvic MRI and was based on investigator-reported measurements according to Response Evaluation Criteria in Solid Tumors (RECIST) guidelines [19]. Toxicity scoring was performed using the Common Terminology Criteria for Adverse Events, Version 4.0 [20]. OS was calculated in months from the date of diagnosis to the first event, including date of the last follow-up or death. Local failure (LF) and distant failure (DF) were defined as the time from diagnosis to local recurrence within the pelvis (LF) and distant metastasis (DF) occurrence. Standard descriptive statistics were used to evaluate the distribution of each factor. Continuous variables were presented as medians and interquartile range, and dichotomous variables were presented as counts and percentages.

The expected number of patients has been calculated according to the Simon's two-stage design. Based on four large randomized phase III trials [21–24], the projected pCR rate after standard preoperative treatment is 15% and an absolute 20% improvement in pCR rate is deemed clinically significant. With an α error of 0.05 and a power of 80%, the planned study would proceed as followed: after a first stage of 8 patients, if three or more patients with a ypT0N0 tumor are observed, accrual of 20 additional patients will be achieved. If this condition is not met, the study will be stopped for futility. After the second step, if there are seven or more ypT0N0 tumor, it can be concluded that the rate of pCR is statistically significantly greater than that of literature.

Statistical analysis was carried out using R-Studio 0.98.1091 software.

## CONCLUSIONS

Our preliminary data supported the continued patients accrual. IC based on FOLFOXIRI regimen combined with targeted agents followed by neoadjuvant CRT and surgical resection is manageable and well tolerated. This combination of therapies will hopefully provide meaningful benefit. Surely, longer follow-up and final results are mandatory to assess if this trimodality strategy consistently translate to improved overall survival and metastasis free survival in LARC patients.
